# A rare cause of infantile achalasia: *GMPPA*‐congenital disorder of glycosylation with two novel compound heterozygous variants

**DOI:** 10.1002/ajmg.a.62859

**Published:** 2022-06-04

**Authors:** Irina Geiculescu, Jason Dranove, Graham Cosper, Andrew C. Edmondson, Eva Morava‐Kozicz, Lauren B. Carter

**Affiliations:** ^1^ Department of Pediatrics Levine Children's Hospital Charlotte North Carolina USA; ^2^ Division of Pediatric Gastroenterology, Hepatology, and Nutrition Levine Children's Hospital Charlotte North Carolina USA; ^3^ Pediatric Surgical Associates, Levine Children's Hospital Charlotte North Carolina USA; ^4^ Division of Human Genetics, Department of Pediatrics Children's Hospital of Philadelphia Philadelphia Pennsylvania USA; ^5^ Department of Clinical Genomics Mayo Clinic Rochester Minnesota USA; ^6^ Department of Pediatrics, Division of Medical Genetics Levine Children's Hospital, Atrium Health Charlotte North Carolina USA

**Keywords:** AAMR, achalasia, congenital disorder of glycosylation, *GMPPA*

## Abstract

Achalasia is rare in the pediatric population and should prompt clinicians to consider genetic disorders associated with this condition. While AAA syndrome (also known as Allgrove or Triple A syndrome) is commonly considered, *GMPPA*‐congenital disorder of glycosylation (CDG) should also be in the differential diagnosis. We report a 9‐month‐old female born to nonconsanguineous parents with achalasia and alacrima found to have two novel compound heterozygous variants in the *GMPPA* gene associated with *GMPPA*‐CDG. This rare disorder is commonly associated with developmental delay and intellectual disability. We discuss management of this disorder including the importance of confirming a genetic diagnosis and summarize reported cases.

## 1 INTRODUCTION

Achalasia is an esophageal motility disorder characterized by absent esophageal body peristalsis and failure of lower esophageal sphincter (LES) relaxation secondary to degeneration of the inhibitory myenteric plexus innervating the LES and esophageal body (Khashab, 2020). This rare disorder typically presents with symptoms of progressive dysphagia, weight loss, and vomiting. Achalasia presents similarly to gastroesophageal reflux, particularly in infants and younger children, which may lead to delays in diagnosis given low index of suspicion for this condition. These delays can lead to failure to thrive, recurrent pneumonia, and repeat hospitalizations.

Achalasia has an incidence as 2.92 per 100,000 in adults (Khashab, 2020), whereas incidence in children is reported at 0.11/100,000 annually (Franklin, 2014), with less than 5% of all achalasia cases presenting before age 16 years (Franklin, 2014). Infantile achalasia is so rare that the incidence in this population is unknown and descriptions are limited to case reports (Franklin, 2014). Although this diagnosis is often isolated and idiopathic in nature, it has been commonly associated with certain diagnoses such as AAA syndrome, which encompasses glucocorticoid insufficiency and ACTH‐resistant adrenal insufficiency, trisomy 21, congenital hypoventilation syndrome, eosinophilic esophagitis, and Chagas disease (Franklin, 2014). Achalasia is also associated with autosomal recessive *GMPPA*‐congenital disorder of glycosylation (CDG), which is due to loss of function variants in the *GMPPA* gene. This rare form of CDG has also been referred to as AAMR syndrome (acronym for alacrima, achalasia, and mental retardation). *GMPPA*‐CDG lacks adrenal insufficiency as a feature which is an important distinction to make from the more commonly thought of AAA syndrome. We present a case of a 9‐month‐old female with achalasia and alacrima with two novel compound heterozygous variants in the *GMPAA* gene, provide a brief review of *GMPPA*‐CDG, and discuss management of this condition.

## 2 MATERIALS AND METHODS

### 2.1 Editorial policies and ethical considerations

This study was approved by the Atrium Health ethics committee. Informed consent was obtained from the family.

## 3 RESULTS

We present a 9‐month‐old female, delivered at 37 weeks gestational age, who was born to nonconsanguineous parents. Mother is of Chinese descent and father's ethnicity is German and Eastern European. She had gastroesophageal reflux, feeding difficulty, and poor weight gain since birth and presented at 2 months of age for gastrointestinal (GI) evaluation. Despite medical intervention including formula changes, histamine‐2 receptor blockers, and proton pump inhibitors, her symptoms persisted. Gagging, coughing, and vomiting within the first few ounces of feeding was concerning for aspiration. The patient was scheduled for a modified barium swallow, which showed oropharyngeal dysphagia and aspiration of thin liquids via standard nipple. An upper GI contrast barium study showed significant esophageal dilation, poor esophageal emptying, lack of peristaltic stripping wave, and a classic “birds beak sign” appearance (Supplemental Information Figure S1). The clinical and radiographic findings were concerning for achalasia.

She was admitted for expedited workup and management. She underwent an upper endoscopy with biopsy showing mild dilation of the esophagus and a subjectively hypertensive LES. Esophageal manometry findings confirmed a diagnosis of type II achalasia. Due to the patient's feeding difficulty and aspiration risk, a gastrostomy tube was placed to ensure proper nutrition while surgical options were discussed. After careful consideration, the decision was made to proceed with a Heller myotomy without fundoplication at 8 months of age.

Due to the unusually young age of diagnosis, referrals to genetics, endocrinology, and ophthalmology were made for evaluation of potential AAA syndrome. Ophthalmology evaluation revealed lack of tears with crying, consistent with alacrima. Endocrine evaluation showed no signs of adrenal insufficiency. Genetics evaluation revealed hypotonia, lack of tearing, and short stature. Subsequent neurology exam also revealed anisocoria, absent pupillary response and torticollis. She is now 9 months old. Although she had mild delayed milestones due to her hypotonia and torticollis, she has made improvements with therapy.

### 3.1 Genetic analysis

Given the concern for AAA syndrome, *AAAS* single gene sequencing was initially sent and was normal. Subsequently, trio exome sequencing was obtained which revealed novel compound heterozygous missense variants of uncertain significance in the *GMPPA* gene; c.874A > C, (p.T292P) which was inherited from her mother and the c.952 C > T, (p.R318W) which was inherited from her father. The c.874A > C variant is not observed in gnomAD, in silico analysis supports that this variant has a deleterious effect on protein structure or function, and the amino acid position T292 is highly conserved. The c.952C > T variant is not observed at a significant frequency in large population cohorts (<0.01% in gnomAD), in silico analysis supports that this variant has a deleterious effect on protein structure or function and the amino acid position R318 is highly conserved. It is also interesting to note that recent cryo‐electron microscopy structural elucidation of the GMPPA‐GMPPB complex and biochemical pull‐down assays described by Zheng et al. implicate residue 318 as important in the interaction between GMPPA and GMPPB. (Zheng et al., 2021) Despite variants being classified as uncertain significance, given her clinical picture, a diagnosis of *GMPPA*‐CDG was made. Her healthy full brother's testing revealed one variant indicating that he is an unaffected carrier.

## 4 DISCUSSION

Although the incidence of achalasia in infants and children is low, it should be included in the differential diagnosis in any infant or child with refractory gastroesophageal reflux, persistent lower respiratory tract infections, and failure to thrive (Diaz, 2020). Even if not specifically ordered to rule out achalasia, a standard upper GI contrast study can assist in making the diagnosis. Overall, it is rare to see the diagnosis in childhood as it usually affects patients in their third to sixth decade of life, rarely presenting prior to twenty  years of age (Chatterjee, 2013).

The first case of infantile achalasia was reported in 1953 in a 6‐month‐old who went on to have a Heller Myotomy at 9 months of age with successful improvement in symptoms (King, 1953). Several case reports since then have described infants as young as 3‐months old diagnosed with achalasia (Franklin, 2014; Moazam, 1976). However, the incidence in the infant population is not known. If diagnosis is suspected, prompt referral to a tertiary or quaternary care center with appropriate subspecialty medical and surgical support is recommended. Once the diagnosis is confirmed, nutrition must be optimized, safety of oral feedings examined closely, and discussion of further management should ensue. In general, the three main options for the definitive treatment of achalasia include Heller myotomy with or without a partial fundoplication wrap, pneumatic dilation, and Peroral Endoscopic Myotomy (POEM) (Wood, 2020; Khashab, 2020). Although medical options such as calcium channel blockers and LES botulinum toxin injection exist, they either have significant side effects and poor efficacy in the former, or only provide temporary relief in the latter (Wood, 2020; Khashab, 2020). There is wide variation in practice worldwide with no agreed upon standard approach in children (van Lennep, 2019). For infants, pneumatic dilation is impractical due to lack of appropriately sized equipment. POEM is a novel technique which has been performed in children, however, the youngest patient reported to date to undergo this technique is 2 years old (van Lennep, 2019). In consultation with local and national experts on the subject, the patient underwent a Heller Myotomy without partial fundoplication at 8 months of age.

Once a diagnosis of achalasia is made, there should be consideration for an underlying genetic diagnosis. Understanding the molecular basis of disease informs management and treatment options. When considering an achalasia diagnosis, many physicians would consider AAA syndrome primarily. AAA syndrome or Triple A Syndrome represents an acronym referring to achalasia, Addison disease, and alacrima (Triple A Syndrome, 2015). It is an autosomal recessive condition due to variants in the *AAAS* gene (Patel, 2017). Given normal genetic testing results in our patient, additional genetic etiologies were considered that are associated with AAA syndrome‐like phenotypes including conditions related to *GMPPA* and *TRAPPC11* genes (Adam, 2018).

*GMPPA* is a gene, which encodes for mannose‐1‐phosphate guanyltransferase alpha, an enzyme which may regulate the GMPPB enzyme involved in the production of N‐linked oligosaccharides (GMPPA Gene‐GDP‐Mannose Pyrophosphorylase, 2022). Loss of function mutations in the *GMPPA* gene have been associated with autosomal recessive N‐linked CDG specifically referred to as *GMPPA*‐CDG. *GMPPA*‐CDG is characterized by intellectual disability and autonomic dysfunction including alacrima and achalasia with onset at birth or in early infancy. Other features can include developmental delay, hypotonia, gait abnormalities, and visual or hearing deficits. There is clinical overlap with AAA syndrome, however, patients with *GMPPA*‐CDG have not been reported with adrenal insufficiency. This is a significant difference between the two conditions and changes ongoing need for endocrine monitoring.

Although a rare disorder, multiple *GMPPA*‐CDG cases have been identified in the literature, which are summarized in Supplemental Information Table S1 (Alabdullatif al., 2016; Gold t al., 2017). Review of 22 cases in total, including our case, revealed 68% of cases are female and 32% are male. All patients had achalasia, 100% had alacrima, and 0% had adrenal insufficiency. All patients had delayed development, intellectual development, and short stature. Ocular symptoms were seen in 63% (12/19), 56% (9/16) had hypotonia, and 46% (6/13) had gait abnormalities. Other features reported include: 36% (5/14) with hearing impairment, 40% (8/20) with decreased sweating, 89% (8/9) with facial dysmorphism, and 40% (2/5) with anodontia. Benitez (2018) reported two patients having new findings of anodontia associated with this disorder. Diaz et al. (2020) reported patients of an indigenous Guatemalan population, suggesting it was a “founder” mutation. The description of dysmorphic features is variable and not all reported patients have had thorough evaluations. Further information can be found in the Supplemental Information Table S1.

Although there are general guidelines regarding management of N‐linked glycosylation disorders, it was challenging to know which surveillance should apply to our patient with *GMPPA*‐CDG. General recommendations were followed including assessing thyroid function, liver function tests, and urinalysis to assess for proteinuria, which were normal. Baseline renal ultrasound was also normal. Given concern for bleeding abnormalities in N‐linked glycosylation disorders, prothrombin time, partial thromboplastin time, and CBC were obtained and normal. Consultation with experts also recommended Factor IX, Factor XI and antithrombin III levels given theoretic risk. These also resulted normal but given pending surgery, surgical team was made aware to have fresh frozen plasma on standby. Our patient had initial endocrine evaluation prior to diagnosis being made and her labs were normal. Given her *GMPPA*‐CDG diagnosis, we will not need to monitor for adrenal insufficiency.

Elevated levels of GDP‐mannose observed in GMPPA‐deficient cells and insight that GMPPA might serve as a regulator of GMPPB led Koehler et al. to speculate that a mannose‐depleted diet might have therapeutic benefit (Koehler et al., 2013). Franzka et al. (2021) provided experimental evidence showing that *GMPPA*‐knock out mice with dietary mannose restriction had improvement in hyperglycosylation, which subsequently prevented neuronal degradation and loss of motor skills. While the body's endogenous production of mannose from glucose exceeds dietary intake (Sharma, 2014) and while not yet of demonstrated benefit in GMPPA‐CDG, additional expert opinion advised avoidance of high‐mannose foods, such as cranberries. Our patient's mother has eliminated cranberries from her diet given she is breastfeeding, although dietary restriction is unlikely to affect mannose content of breastmilk. Given normal initial evaluations, long‐term management will focus on ophthalmology, gastroenterology, and development. The diagnosis has given the family access to research opportunities, including an ongoing natural history study (Clinical and Basic Investigations Into Congenital Disorders of Glycosylation, ClinicalTrials.gov Identifier: NCT04199000) and a clinical trial regarding dietary monosaccharide supplementation (Dietary Monosaccharide Supplementation in Patients With Congenital Disorders of Glycosylation, ClinicalTrials.gov Identifier: NCT04198987). Given the recent additional evidence of benefit from mannose restriction, a clinical trial evaluating clinical outcomes with mannose restriction should also be a priority. Identifying a genetic diagnosis in this family has allowed for tailored management, potential treatment opportunities, as well as recurrence risk counseling.

## 5 CONCLUSION

We report a 9‐month‐old term female born to nonconsanguineous parents with achalasia and alacrima found to have two novel compound heterozygous variants in the *GMPPA* gene associated with *GMPAA*‐CDG, a rare type of CDG. We summarize previously reported cases, provide a brief review of *GMPPA*‐CDG, and discuss management of this condition. A high index of suspicion is needed to diagnose achalasia in infants, and once a diagnosis is made it is paramount to pursue genetic testing to confirm a diagnosis and guide clinical management.

## AUTHOR CONTRIBUTIONS

All persons who are listed as authors certify that they have participated and take responsibility for the content of the manuscript.

Irina Geiculescu: conceptualization, writing‐original draft preparation. Lauren Carter: supervision, writing‐review, and editing. Jason Dranove: supervision, writing‐review, and editing. Graham Cosper: investigation, validation, visualization. Andrew C. Edmondson: writing‐review and editing. Eva Morava‐Kozicz: writing‐ review and editing.

## CONFLICT OF INTEREST

The authors have no conflicts of interest to disclose.

## Supporting information


**FIGURE S1** MBSS showcasing tapering of the inferior esophagus

**TABLE S1** Summary of GMPPA‐CDG cases in literature

## Data Availability

The data that support the findings of this study are available from the corresponding author upon reasonable request.
