# Supplementary material for: A rare cause of infantile achalasia: GMPPA‐congenital disorder of glycosylation with two novel compound heterozygous variants
Source: Am J Med Genet A. 2022 Jun 4;188(8):2438–42. doi: 10.1002/ajmg.a.62859 (PMC9283290; doi:10.1002/ajmg.a.62859)
Supplement: Supplementary file 1 — FIGURE S1 MBSS showcasing tapering of the inferior esophagus [file AJMG-188-2438-s001.docx]

Outpatient upper GI and modified barium swallow study (MBSS) showcased significant esophageal dysmotility with overall concerns for achalasia with classic birds beak sign appearance as noted in **Figure 1**.


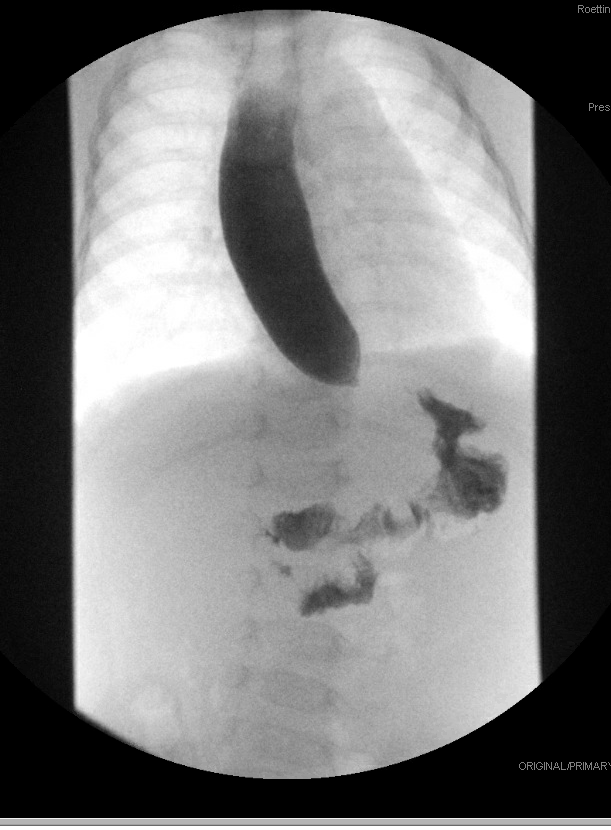


Figure 1: MBSS showcasing tapering of the inferior esophagus
